# Enhanced In Vitro Plant Morphogenesis of Tobacco: Unveiling Indoleamine-Modulated Adaptogenic Properties of Tulsi (*Ocimum sanctum* L.)

**DOI:** 10.3390/plants13101370

**Published:** 2024-05-15

**Authors:** Vanessa Vongnhay, Mukund R. Shukla, Murali-Mohan Ayyanath, Karthika Sriskantharajah, Praveen K. Saxena

**Affiliations:** Department of Plant Agriculture, Gosling Research Institute for Plant Preservation, University of Guelph, Guelph, ON N1G 2W1, Canada; vvongnha@uoguelph.ca (V.V.); mshukla@uoguelph.ca (M.R.S.); ayyanath@uoguelph.ca (M.-M.A.); sriskank@uoguelph.ca (K.S.)

**Keywords:** adaptogen, N-acetyl-serotonin, shoot organogenesis, somatic embryogenesis, tryptamine, holy basil, tulsi

## Abstract

The medicinal plant tulsi (*Ocimum sanctum* L.) is acknowledged for its invigorating and healing properties that enhance resilience to stress in various human and animal models by modulating antioxidant compounds. While extensive research has documented these effects in humans, the adaptogenic potential of tulsi in stressful in vitro plant systems has not been explored. This study aimed to elucidate the adaptogenic properties of tulsi leaf extract on the in vitro regeneration of tobacco leaf explants through an investigation of the indoleamines at different developmental stages. Shoot regeneration from leaf explants on the medium supplemented with tulsi extract (20%) was compared to the control, and the differences in indoleamine compounds were analyzed using ultra-performance liquid chromatography. Treatment of the explants with the extract resulted in an almost two-fold increase in the number of regenerants after four weeks of culture, and 9% of the regenerants resembled somatic embryo-like structures. The occurrence of browning in the extract-treated explants stopped on day 10, shoots began to develop, and a significant concentration of tryptamine and N-acetyl-serotonin accumulated. A comparative analysis of indoleamine compounds in intact and cut tobacco leaves also revealed the pivotal role of melatonin and 2-hydroxymelatonin functioning as antioxidants during stress adaptation. This study demonstrates that tulsi is a potent adaptogen that is capable of modulating plant morphogenesis in vitro, paving the way for further investigations into the role of adaptogens in plant stress biology.

## 1. Introduction

The term ‘adaptogen’ was originally used to describe a class of pharmacologically active compounds that induce a generalized increase in resistance in mammals, enabling them to cope with stressors and adapt to exceptional strains [1,2]. Later, the term ‘adaptogen’ was extended to describe a group of plants that exhibit adaptogenic properties. To qualify as an adaptogen, a plant must demonstrate specific characteristics, including the ability to relieve and minimize stress-induced damage [3]. Plants such as ashwagandha, St. John’s Wort, ginseng, ginkgo, and tulsi have a long history of utilization in traditional Ayurvedic and Chinese medicines, spanning several centuries. These medicinal plants are esteemed for their therapeutic potential in addressing various health conditions, including infectious diseases and mental health disorders such as depression, anxiety, and chronic stress [4,5,6]. These plants are also known for their significant antioxidant and anticarcinogenic properties, which are primarily due to a diverse range of biologically active chemical compounds. Such compounds function as potent scavengers of reactive oxygen species (ROS) and reactive nitrogen species (RNS), which are known to inflict damage upon vital biomolecules such as DNA, proteins, cells, and the tissues of multiple organs [7,8,9]. Consequently, the plants with demonstrated abilities to enhance cellular defense mechanisms and combat oxidative and nitrosative stresses are considered as ‘adaptogens’ within the field of natural medicine.

Tulsi (*Ocimum sanctum*), referred to by more than 350 local names, including holy basil, is regarded as the ‘Queen of Herbs’, owing to its multifaceted capabilities in addressing a wide array of stressors and health concerns in both humans and animals. These beneficial effects are attributed to the broad range of secondary metabolites present within the plant, more specifically in the leaves [10,11,12]. In a parallel vein, plants themselves encounter various stressors, stemming from adverse environmental conditions, nutritional deficiencies, and developmental challenges [13,14]. Few studies have explored the potential of medicinal plants to ameliorate stress in plants grown in vitro. For example, Neves et al. [15] utilized Brazilian ginseng (*Pfaffia glomerata*) to investigate the cytogenotoxic effects on *Allium cepa* cells. However, to the best of our knowledge, utilization of adaptogenic plants such as tulsi for mitigating stresses in plants has not been studied. In vitro culture and regeneration steps exert significant stress during the processes of shoot and root organogenesis and somatic embryogenesis [16,17]. These developmental events involve the induction of plant somatic cells to form specialized structures, passing through critical stages of morphogenesis including induction, histodifferentiation, maturation, and conversion into plantlets [18]. Throughout these stages of development, regenerating explants are exposed to multiple nutritional and environmental stresses that affect their physiology and morphogenetic outcomes [16].

Stress mitigation is commonly linked to the scavenging of ROS through both enzymatic and non-enzymatic antioxidant defense systems. Several antioxidant compounds have been identified in tulsi, including ocimumoside A and B and melatonin (MEL) [19,20,21]. Indoleamines are an example of non-enzymatic antioxidant defense systems and play a pivotal role in enabling plants to redirect their growth and development in response to various stresses, including those associated with morphogenesis in vitro [22,23]. The structural backbone of the indoleamine pathway is tryptophan (TRP), an essential amino acid, which contributes to de novo shoot organogenesis in the culture of St. John’s Wort [24]. Further down this pathway, the precursor molecules tryptamine (TRM) and 5-hydroxytryptophan (5-HTP) are synthesized from TRP through the processes of decarboxylation and hydroxylation, respectively [25,26]. The neurotransmitter serotonin (SER) is subsequently synthesized from TRM and 5-HTP [27], leading to the production of N-acetyl-serotonin (NAS) [28]. The NAS is then converted to MEL [29]. MEL is known to be unstable and rapidly converts into its metabolites, including 2-hydroxymelatonin (2-OHMEL) and N(1)-acetyl-N(2)-formyl-5-methoxykynuramine (AFMK) [30]. The majority of the studies conducted thus far have primarily focused on SER and MEL as two potent antioxidants involved in mitigating stress in plant systems. These metabolites have also been implicated as inducers of in vitro root and shoot organogenesis as well as somatic embryogenesis [31,32]. However, to the best of our knowledge, the roles of other metabolites within the indoleamine pathway in the context of plant regeneration in vitro have yet to be elucidated.

In this regard, we hypothesized that the addition of tulsi leaf extract to the tissue culture medium would improve in vitro organogenesis and somatic embryogenesis in the model plant *Nicotiana tabacum*, possibly by influencing the modulation of indoleamine metabolites. Hence, our study was designed with the following objectives: (1) to discern the effects of an aqueous extract of tulsi on plant morphogenesis using an in vitro regeneration model of *N. tabacum*; and (2) to unravel the intricate interplay among metabolites within the indoleamine pathway and the observed regeneration phenomena.

## 2. Results

The leaf explants were cultured on regeneration medium with and without tulsi leaf extract and observed for signs of stress, including browning, the appearance of necrotic tissue, and regeneration from the cultured explants [33].

### 2.1. Effects of the Tulsi Leaf Extract on the Browning of Leaf Explants

**Figure 1:**
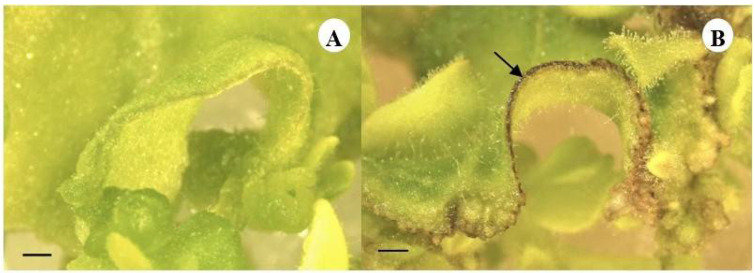
The leaf disc explants cultured on the control medium displaying the absence of browning (**A**) compared to the leaf explants supplemented with tulsi leaf extract (20%) showing browning (arrow) on the edges after 10 d of culture (**B**) (bar = 1.0 mm).

The leaf disc explants that were cultured on the control medium without tulsi leaf extract remained green, displaying no signs of browning throughout the culture duration (Figure 1A). Conversely, the explants cultured in the medium containing tulsi leaf extract initially showed browning along the edges of the leaf disc (Figure 1B). Notably, the browning of the leaf edges ceased its progression after 10 d of culture, coinciding with the ongoing growth of the explants and the initiation of shoot regeneration. The shoot buds developed from the periphery of the explant and progressed into mature shoots between 15 and 20 d periods. In contrast, the control plants did not exhibit any signs of browning, enabling the observation of shoot bud development at an earlier stage, occurring between days 5 and 10.

### 2.2. Effects of Tulsi Leaf Extract on In Vitro Regeneration of N. tabacum

In the preliminary experiments with *N. tabacum*, three different concentrations of tulsi leaf extract (1, 10 and 20%) were added to the tissue culture medium. The treatment with the 20% concentration resulted in a significantly higher number of shoots as well as structures resembling somatic embryos (Table S1). Enrichment of the culture medium with tulsi leaf extract at concentrations higher than 20% did not enhance regeneration and resulted in greater toxic effects due to the presence of phenolic compounds in the extract. Consequently, for all further experiments, 20% tulsi leaf extract was selected for evaluating its adaptogenic potential in enhancing in vitro regeneration of *N. tabacum* leaf discs. The application of 20% tulsi leaf extract significantly (53%; *p* < 0.0001) increased the production of regenerants in the form of shoots and embryo-like structures (Table 1) in comparison to the control group. Moreover, after a treatment period of 4 weeks, the treated explants exhibited a 36% greater production of organogenic shoots, measuring over 1 cm in length (*p* < 0.0001), and a 100% increase in the number of embryo-like structures at the globular and subsequent developmental stages (*p* < 0.0001). In contrast, the control plants predominantly produced shoots.

**Figure 2:**
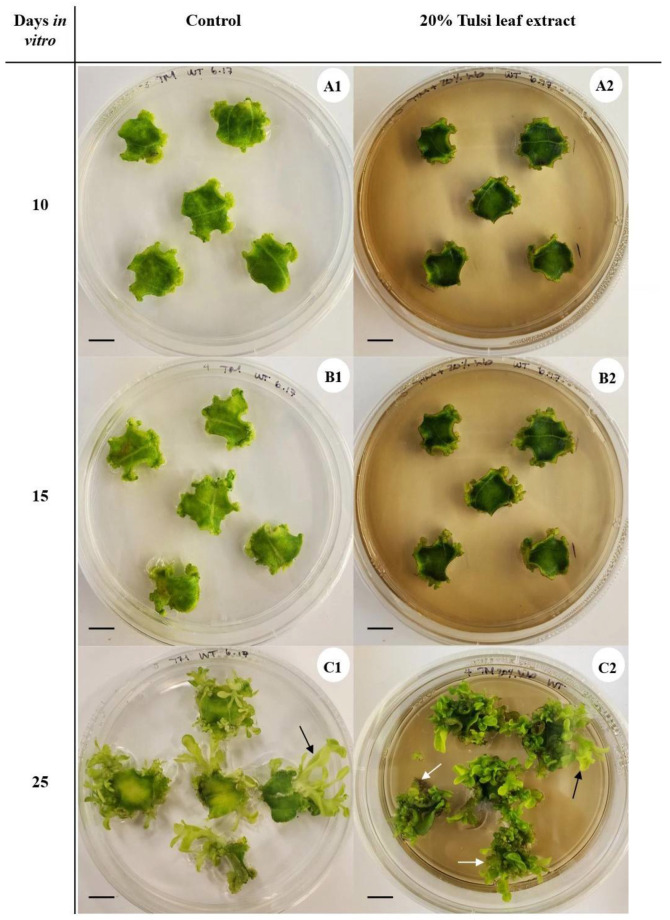
Developmental progression of leaf disc explants of *N. tabacum* in the medium without tulsi leaf extract (control) and the medium containing 20% tulsi leaf extract after 10 (**A1**,**A2**), 15 (**B1**,**B2**), and 25 (**C1**,**C2**) d of in vitro culture, displaying the emergence of shoots (black arrows) and somatic embryo-like structures (white arrows). Bar = 1.0 cm.

Visual observations showed substantial differences in the organogenesis between the control group and the group subjected to tulsi leaf extract across various in vitro developmental stages. From day 0 to day 5, the explants from both treatment groups exhibited an increase in size. However, from day 5 to 10, the control group explants displayed the formation of friable callus and initiation of shoot buds along the edges of the explants (Figure 2(A1)), whereas the explants treated with the tulsi extract visibly commenced callus and shoot bud initiation only after 10 d (Figure 2(A2)). Subsequently, the explants in both treatment groups developed shoots (Figure 2B). Nevertheless, the control group produced fewer but normal shoots per explant (Figure 2(C1)), whereas the extract-treated group produced a significantly greater number of smaller shoots, approximately 1 cm in length, as well as somatic embryo-like structures (Figure 2(C2)).

### 2.3. Histology of Shoot Organogenesis and Somatic Embryogenesis

**Figure 3:**
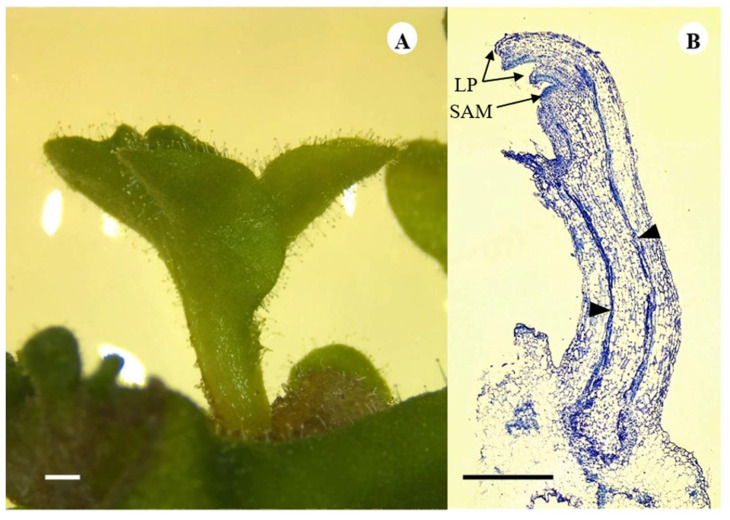
Shoot development in the medium containing 20% tulsi leaf extract observed after 15 d of culture of *N. tabacum* leaf discs (**A**). Longitudinal section of the shoot showing the leaf primordia (LP) and the shoot apical meristem (SAM) as well as the provascular tissue (triangles) (**B**) (bar = 1.0 mm).

To determine the development of shoots from leaf explants cultured under control conditions and in the medium supplemented with 20% tulsi leaf extract, histology of the regenerating explants was conducted at 5, 10, 15, and 25 d of culture. This analysis unveiled the presence of provascular tissue connecting the explant to the developing shoot, a characteristic mode of organogenic shoot development (Figure 3A,B). Furthermore, discernible features such as the apical meristem and leaf primordia were also observed.

**Figure 4:**
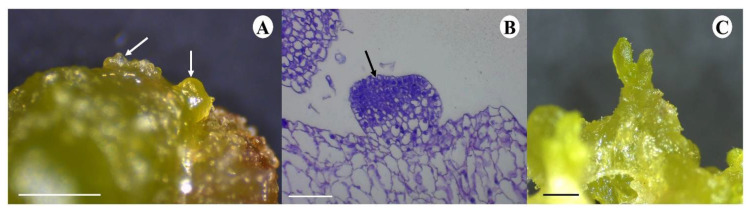
*N. tabacum* leaf disc regeneration in the medium containing 20% tulsi leaf extract after 20 d of culture. Somatic embryo-like structures in a heart shape stage (white arrows) on the leaf explant surface (bar = 1.0 mm) (**A**). Longitudinal section showing the meristematic tissue (**B**) (black arrow, bar = 0.1 mm) and the heart-shaped stage transitioning to the torpedo stage (**C**) (bar = 1.0 mm).

Similarly, discernible somatic embryo-like structures were observed emerging from the callus after 20 d of culture. However, the emergence of these structures were not uniform, with varying stages of development simultaneously observed at a single time point, as depicted in Figure 4A,C. Histological analysis revealed prominently darkly stained apical meristematic tissue visible in the heart-shape stage developing along the periphery of the explant (Figure 4B).

### 2.4. Effects of Tulsi Leaf Extract on the Indoleamine Pathway

#### 2.4.1. Whole Leaf vs. Leaf Disc on Day 0

**Figure 5:**
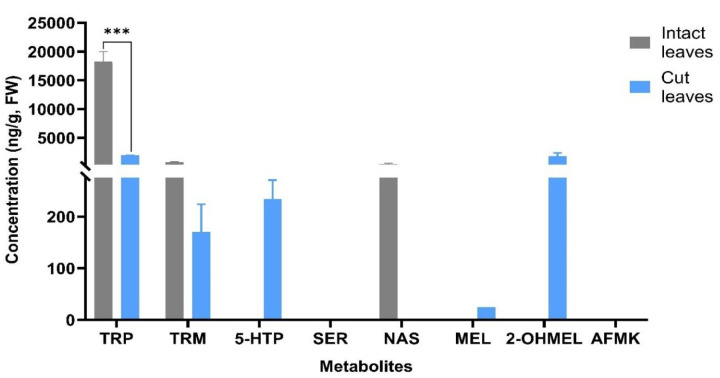
The concentration of the metabolites (tryptophan (TRP), tryptamine (TRM), 5-hydroxytryptophan (5-HTP), serotonin (SER), N-acetyl-serotonin (NAS), melatonin (MEL), 2-hydroxymelatonin (2-OHMEL), and N(1)-acetyl-N(2)-formyl-5-methoxykynuramine (AFMK)) in *N. tabacum* leaf tissues that were either intact whole leaf or cut leaf discs (day 0). Data represent the means ± SE of two biological replicates and three technical replicates. *** Indicates significant differences at *p* < 0.0001 between the intact and cut tobacco leaves based on Tukey’s HSD at α = 0.05.

Indoleamine pathway metabolites, including TRP, TRM, 5-HTP, SER, NAS, MEL, 2-OHMEL, and AFMK, were analyzed in intact whole-leaf samples as well as cut leaf disc explants of *N. tabacum*. Among these indoleamines, TRP, TRM, and NAS were detected in the intact leaves, while all the other indoleamine metabolites remained undetectable, presumably residing below the detection limit of the UPLC-MS instrumentation (Figure 5). It is noteworthy that the TRP and NAS concentrations were significantly higher in the intact leaves, which were devoid of any wounding/stress process. A particularly distinctive finding was the exclusive detection of NAS in the intact leaves, with a concentration of 417 ng/g fresh weight (FW). In contrast, the TRP concentration in the cut leaves was significantly lower, with an 89% reduction (*p* < 0.0001) compared to the intact leaves. TRM, the downstream molecule of TRP, was detected in both the intact and cut leaves at similar concentrations. Notably, further downstream molecules such as 5-HTP, MEL, and 2-OHMEL were detected only in the cut leaves with concentrations of 234 ng/g FW, 25 ng/g FW, and 1827 ng/g FW, respectively, while neither intact nor cut leaves displayed SER or AFMK in the analyzed samples.

#### 2.4.2. Indoleamines in Tulsi Leaf Extract

**Figure 6:**
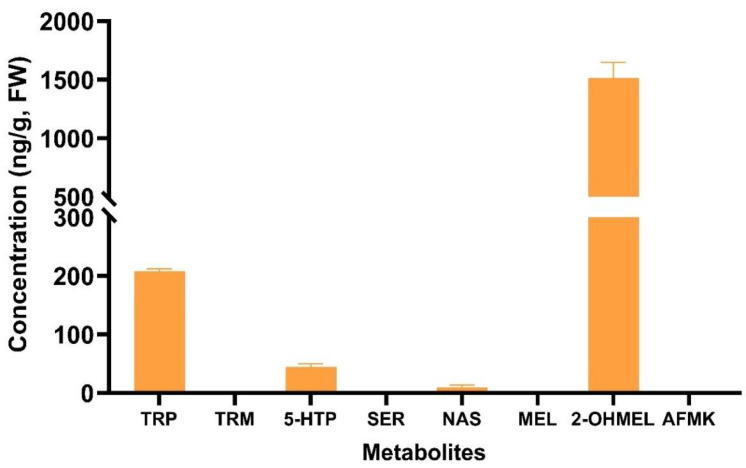
The fresh weight concentration of various indoleamine metabolites (tryptophan (TRP), tryptamine (TRM), 5-hydroxytryptophan (5-HTP), serotonin (SER), N-acetyl-serotonin (NAS), melatonin (MEL), 2-hydroxymelatonin (2-OHMEL), and N(1)-acetyl-N(2)-formyl-5-methoxykynuramine (AFMK)) in the tulsi leaf extract. Data represent the means ± SE of two biological replicates and three technical replicates measured in the pure tulsi leaf extract.

Among the indoleamines, TRP, 5-HTP, NAS, and 2-OHMEL were detected in the pure tulsi leaf extract, whereas all the other compounds (TRM, SER, NAS, MEL, and AFMK) remained undetectable. Presumably, these were present below the detection limit of the UPLC-MS instrumentation (Figure 6). 2-OHMEL, a prominent melatonin metabolite, was quantified at an approximate concentration of 1500 ng/g FW. This concentration was nearly 6 times greater than that of TRP and approximately 15 times higher than the concentration 5-HTP (Figure 6). NAS was also detected in the extract, albeit in a minimal quantity (approximately 10 ng/g FW).

#### 2.4.3. Effects of Tulsi Leaf Extract during Culture Duration

**Figure 7:**
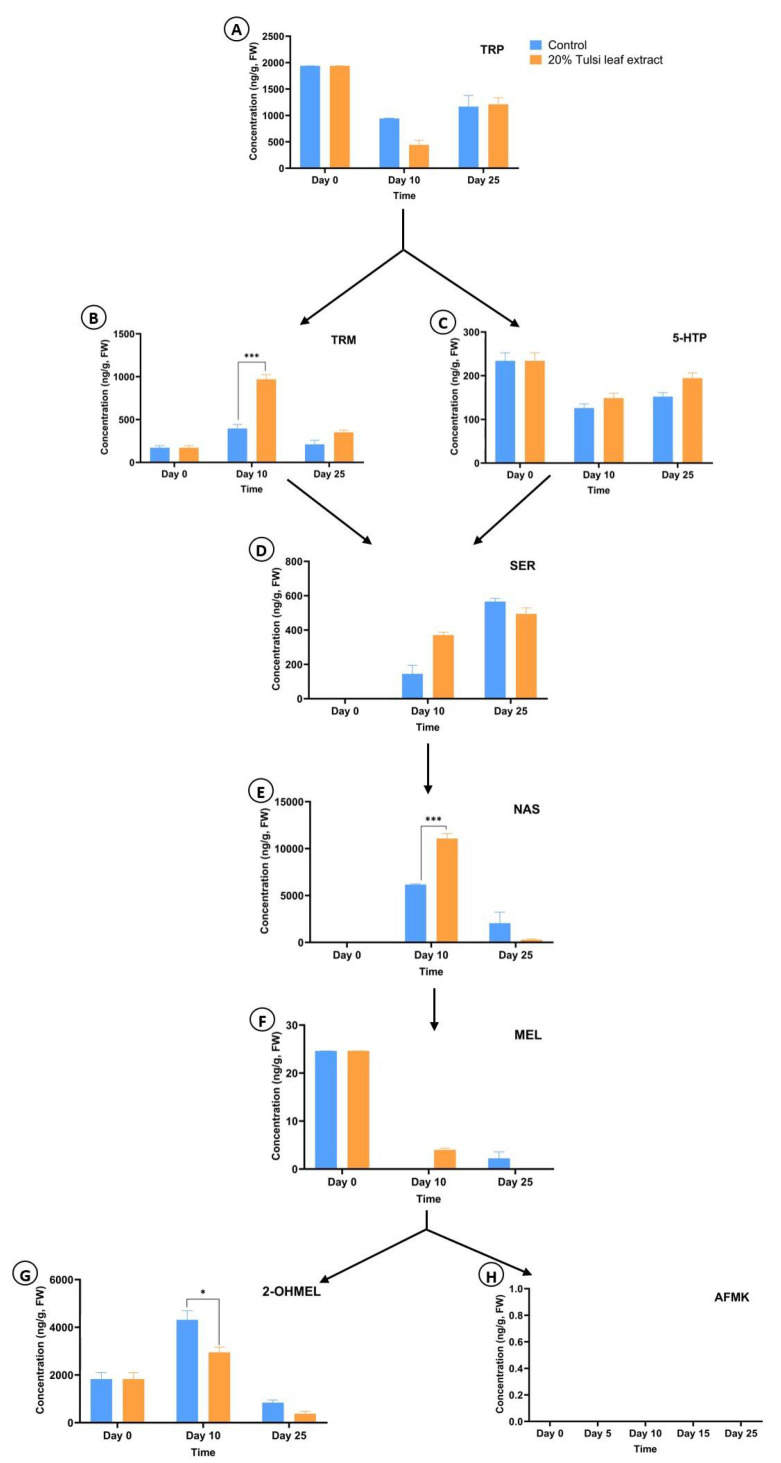
The concentrations of the compounds present in the indoleamine pathway, tryptophan (TRP) (**A**), tryptamine (TRM) (**B**), 5-hydroxytryptophan (5-HTP) (**C**), serotonin (SER) (**D**), N-acetyl-serotonin (NAS) (**E**), melatonin (MEL) (**F**), 2-hydroxymelatonin (2-OHMEL) (**G**), and N(1)-acetyl-N(2)-formyl-5-methoxykynuramine (AFMK) (**H**), in *N. tabacum* leaf discs at 0, 10, and 25 d of culture on the medium supplemented with 20% tulsi leaf extract. Data represent the means ± SE of two biological replicates and three technical replicates of each treatment and time point. * Indicates significant differences at *p* < 0.05; *** indicates significant differences at *p* < 0.0001 between the control and tulsi leaf extract at each time point based on Tukey’s HSD at α = 0.05.

In general, the concentrations of all the metabolites (TRP, TRM, 5-HTP, SER, NAS, MEL, 2-OHMEL, and AFMK) were found to be different in the treated explants compared to the control explants over the culture duration at 0, 10, and 25 d of culture (Figure 7). TRP was consistently detected at all time points in both treatment groups, although the differences between treatments at the same time point were not statistically significant. In the control group, TRP was present at its highest concentration on day 0 (1938 ng/g FW) but subsequently declined to 940 ng/g FW on day 10 before slightly increasing to 1166 ng/g FW on day 25 (Figure 7A). The tulsi leaf extract group followed the same trend, with TRP levels decreasing from day 0 (1938 ng/g FW) to 442 ng/g FW on day 10, followed by a slight increase (1209 ng/g FW) over that detected in the control group on day 25.

TRM was quantified in all the time points in both treatment groups; however, on day 10, the TRM concentration was 59% higher in the treatment group (*p* < 0.0001) than in the control. Importantly, this coincided with the time that regeneration began to appear (Figure 1). In the control group, TRM increased 50% from day 0 (170 ng/g FW) to 10 (394 ng/g FW), followed by a subsequent decrease to 210 ng/g FW on day 25. Similarly, the extract-treated group followed a comparable trend to the control, with an 82% increase in the TRM concentration from day 0 to 10 (968 ng/g FW), followed by a decline to 25% of its day 10 concentration, 350 ng/g FW on day 25 (Figure 7B). Notably, on day 25, the TRM level was higher in the treated explants than in the control; however, the difference was not significant.

Concurrently, 5-HTP was highest on day 0 (234 ng/g FW) and then decreased by 46% on day 10 (126 ng/g FW) and increased slightly on day 25 (152 ng/g FW) in the control group. Similarly, the concentration in the treated explants decreased on day 10 (149 ng/g FW) and slightly increased on day 25 (194 ng/g FW) (Figure 7C). However, the differences between the treatment and control groups were not significant during the growth period.

In contrast to the preceding metabolites, SER was non-detectable on day 0 and followed an upward trend throughout day 10 and 25 in both treatment groups. In the control, SER increased from 145 ng/g FW on day 10 to 566 ng/g FW on day 25, marking a 74% increment (Figure 7D). Similarly, the concentration of SER in the tulsi leaf extract group increased consistently as the culture period progressed (day 10 = 370 ng/g, day 25 = 494 ng/g FW). On day 10, the SER in the extract-treated group was 60% higher than in the control group (*p* < 0.0527), coinciding with the onset of regeneration events at that specific time point (Figure 1).

Similar to SER, NAS was also not detected on day 0 but increased to its peak concentration on day 10 in both treatment groups when regeneration appeared. Notably, on day 10, a significantly higher concentration of NAS (44%) was detected in the tulsi leaf extract group than in the control group (*p* < 0.0001). Subsequently, in the control group, the NAS level decreased to 2046 ng/g FW, while in the extract group, the NAS concentration decreased to 274 ng/g FW (Figure 7E).

MEL was present at its highest concentration on day 0, measuring 25 ng/g FW, but was undetectable on day 10 in the control group. It re-emerged on day 25, albeit at a lower concentration of 2.2 ng/g FW. In contrast, the concentration in the tulsi leaf extract group exhibited a different pattern, with MEL detected on day 10 (4.0 ng/g FW) but not on day 25 (Figure 7F).

2-OHMEL was consistently present at all the time points and maintained a higher concentration than that observed for MEL. In the control plants, it increased by 58% from day 0 to 10, coinciding with the onset of regeneration in culture, before decreasing to its lowest concentration on day 25 (842 ng/g FW). Similarly, the extract-treated plants followed the same trend, reaching their peak concentration on day 10 (2948 ng/g FW). However, at this time point, the concentration of 2-OHMEL in the treated plants was significantly lower than that in the control group (*p*< 0.004). Furthermore, it declined to its lowest concentration of 376 ng/g FW on day 25 (Figure 7G). Notably, AFMK was not detected in the control or the treated explants across any of the time points (Figure 7H).

## 3. Discussion

The adaptogenic properties of tulsi have been extensively proven in clinical studies involving humans, highlighting its effectiveness in treating numerous ailments [10,12]. Natural health products are often sought after when synthetic medicines fail to prove effective in ameliorating or preventing the underlying problems [3]. Such functions of tulsi are attributed to its broad range of phenolic compounds, including numerous essential oils, terpenes, and flavonoids [34,35]. Similar to human stressors, plants cultivated in vitro also face a myriad of stressors. However, to the best of our knowledge, the utilization of tulsi, a highly effective adaptogen plant, has not yet been explored for the modulation of plant morphogenesis in vitro.

The scope of this study was to elucidate the role of tulsi leaf extract in modulating plant morphogenesis potentially mediated by the interplay of various indoleamine compounds. We used *N. tabacum* leaf explants as the experimental system because of the consistency of regenerative responses required for phytochemical analysis. The use of tulsi leaves as the source of explant would interfere with and influence the effects of the extract on regeneration due to their high phenolic content [36,37,38,39]. Our data demonstrated a significant improvement in the regeneration of *N. tabacum* explants following the addition of tulsi extract to the culture medium (Table 1). Furthermore, somatic embryo-like structures were observed exclusively in the extract-treated explants, which were entirely absent in the control group (Table 1). Organogenesis represents the most common type of regeneration in plant tissue cultures, whereas the transition to an altered route of morphogenesis such as somatic embryogenesis necessitates specific signals and stressors for initiation [40]. These stressors are often in the form of plant growth regulators (PGRs) such as thidiazuron (TDZ) [41] and 2,4-dichlorophenoxyacetic acid (2,4-D) [42].The benefits of natural products on development and regeneration have been observed using *Aloe vera* and seaweed extracts [43,44]. Extracts of these species induced a greater abundance of shoots, leaves, and roots, along with increased height and weight in aspen [43], and improved the production and maturation of tomato somatic embryos [44].

In addition to the increased regenerants observed in the treatment with 20% tulsi extract, the notable occurrence of browning at the edges of the explant was a distinguishing factor observed specifically at the beginning of the regeneration period. The explants treated with the extract exhibited progressive browning along the edges until day 10, at which point regeneration began (Figure 1). In contrast, the control group showed no such browning and developed shoot buds between days 5 and 10 in culture (Figure 1B). The occurrence of browning in cultures can be linked to the accumulation and subsequent oxidation of phenolic compounds present within the explant [19,45]. Furthermore, the additional stress induced by these phenolic compounds likely contributed to the formation of somatic embryo-like structures [46,47]. It is also noteworthy that the prevention of further browning in the explants treated with the extract may be associated with the presence of indoleamines, specifically SER and MEL, which have a high antioxidant potential [48,49].

This antioxidative role of indoleamine metabolites was further elucidated by comparing the indole metabolites of intact and cut *N. tabacum* leaves on day 0 (Figure 5). The significantly greater concentration of TRP in the intact leaves may suggest TRP as a storage molecule until a stressor signal triggers its conversion into downstream molecules [50]. This is also confirmed by the lower levels of TRP in the cut leaves and the concurrent production of downstream molecules 5-HTP, MEL, and 2-OHMEL. Furthermore, the absence of NAS in the cut leaves could indicate the role of NAS as a storage molecule until its conversion into MEL, eventually forming its more stable product, 2-OHMEL. Both MEL and 2-OHMEL play essential roles in mitigating stress and counteracting the effects of associated ROS molecules [51,52,53]. Additionally, the enzyme N-acetylserotonin methyltransferase (*ASMT*), responsible for catalyzing *O*-methylation of NAS into MEL, has been found to be induced by various stressors [54]. Therefore, the presence of NAS in the intact leaves is likely due to the lack of expression of the *ASMT* gene in the absence of stress [55,56].

The leaf extract contained TRP, 5-HTP, NAS, and 2-OHMEL (Figure 6). The presence of 2-OHMEL as the most abundant metabolite (approx. 1500 ng/g FW) suggests the conversion of MEL and SER present in tulsi leaves into 2-OHMEL, which is the dominant melatonin metabolite and is suggested to be involved in mitigating multiple concurrent stresses [52,57]. Several studies have noted the significant difference in concentration between MEL and 2-OHMEL, suggesting that 2-OHMEL is produced at a 300-fold higher rate than that of MEL in the presence of both endogenous and exogenously applied MEL [51]. Additionally, the presence of 5-HTP has primarily been reported in the fruits and seeds of various species [58,59]. This suggests that our study may be the first to document the presence of 5-HTP, as well as NAS and 2-OHMEL, in the leaf extract.

The interaction between the indoleamines of the extract and the explants revealed significant roles of the indoleamine pathway in the regeneration of *N. tabacum* explants (Figure 7). TRP appears to be one of the key molecules involved in these regeneration events, as its concentration on day 0 was the highest of all time points (Figure 7A). Earlier, Erland and Saxena [24] observed that the presence of TRP for only 24 h significantly increased de novo shoot organogenesis in a culture of St. John’s wort, suggesting that TRP is involved in the signaling of numerous pathways that often use it as a precursor. Supplementation with tryptophan increased regeneration in seed cultures of *Oryza sativa* through somatic embryogenesis [60] and enhanced growth and development in *Catharanthus roseus* [61] and *Hypericum perforatum* [62], providing further evidence of its importance in modulating morphogenesis.

In the present study, TRM peaked on day 10, significantly more so in the extract group, indicating its pivotal role in the induction of morphogenesis, as shoot development began at this point (Figure 7B). Commisso et al. [25] observed an increase in the TRM concentration during the induction and development of young tomato leaves, likely acting as an insect deterrent. The increase in TRM on day 10 could act as a precursor for IAA, a key auxin involved in the induction of organogenesis [63,64]. It is also notable that a higher concentration of TRM was present in the leaf tissues. Furthermore, TRM contributes to the production of TRM-linked downstream molecules [50]. Higher concentrations of TRM also increased TRM-linked indolyl metabolites through the modulation of the WsWRKY transcription factor in ashwagandha, a potent adaptogenic plant [65]. Our results also support these findings, as increased levels of TRM resulted in a higher concentration of SER, identifying TRM as a key molecule for the downstream biosynthesis of antioxidants (Figure 7B).

Concurrently, 5-HTP is an alternate metabolite that is responsible for the synthesis of SER. The 5-HTP molecule was present in lower concentrations than that of TRM, with its highest concentration found on day 0 and subsequently decreasing on day 10 (Figure 7C). This suggests that 5-HTP may be synthesized into SER. The addition of 5-HTP to the growth medium led to 5-fold increase in the concentration of SER in tomato seedlings [66], and 5-HTP has been utilized as a supplement to counter serotonin-related disorders [67]. Additionally, reports of 5-HTP in the root allelochemical exudates of numerous species suggest a potential role of 5-HTP in defense signaling or as a deterrent [67]. The observed increase in 5-HTP in our experiments with *N. tabacum* on day 0 after the leaf discs were cut supports the possibility of a protective role of 5-HTP.

Erland et al. [68] speculated that SER may interact with auxin gradients, gene expression, and signaling networks, as the promotion of shoot production was often linked to the inhibition of root growth in cultures of plants such as *Hypericum perforatum* [62]. Our data confirm these speculations, as the regeneration of shoots and embryo-like structures occurred at the same time points when the SER levels increased (day 10 and 25) (Figure 7D). Furthermore, the increase in SER content from non-detectable on day 0 to 370 ng/g FW in the treatment group is potentially linked to its antioxidant potential, with an enhanced ability to scavenge ROS preventing the oxidation of the phenolic compounds and the progression of browning [46,61].

NAS, similar to SER, has been shown to display a high antioxidant potential, more so than that of MEL in lymphocytes [69], and this antioxidant activity is suggested to work independently of MEL [70]. Our results suggest that NAS also plays a vital role as a stable stress-busting molecule, regulating stress within the developing explant [71,72]. A significant increase in NAS concentration was observed in the explants treated with tulsi leaf extract on day 10 (Figure 7E), where it was approximately double that of the control. This increase corresponds to the observed inhibition of further browning on day 10 (Figure 4), as well as to the induction of shoots, resulting in a higher abundance of shoots compared to the control (Table 1).

MEL, another potent antioxidant, was observed in high concentrations on day 0 but remained low in both the extract and control groups on days 10 and 25, respectively (Figure 7F). As stated previously, the higher concentration of MEL on day 0 is linked to its role as an antioxidant in the presence of stressors such as tissue wounding [73]. Furthermore, the presence of MEL on day 10 in the treated group may suggest its role in the prevention of the oxidation of phenolic compounds, thus preventing browning, as well as its role as an inductive signal of regeneration [23,48]. MEL has been reported extensively to modulate and induce root and shoot organogenesis in numerous species [32,53], as well as somatic embryogenesis in *Coffea canephora* [74]. The balanced interactions between MEL and SER in regulating plant morphogenesis and development are similar to those observed between auxin and cytokinins [68].

The presence of the melatonin metabolite 2-OHMEL was significant between the control and extract-treated explants on day 10, which suggests an increased production of endogenous 2-OHMEL in the control. The 2-OHMEL in the extract was presumably utilized more rapidly to minimize the stressors present in the extract-treated explants, increasing regeneration, as the resources were no longer directed to combat these stresses. Multiple studies have reported tolerance conferred by applied 2-OHMEL against a variety of stressors including cold and drought stress in several crop species, as well as an increase in endogenous 2-OHMEL in response to numerous concurrent stressors [51,75,76].

The melatonin metabolite AFMK was not detected in either treatment at any time point. Hence, it is possible that AFMK is either not synthesized or is present in non-detectable quantities in tobacco tissues growing in a culture environment in vitro. AFMK has only been detected in water hyacinth [77] and rice [78], and its biosynthesis may be plant- or stress-specific. Overall, our observations demonstrate the interplay between indoleamines such as TRP, 5-HTP, NAS, and 2-OHMEL during *N. tabacum* regeneration under the influence of tulsi extract by minimizing stress and modulating morphogenesis.

This study presents the first evidence that tulsi leaf extract can act as a potent modulator of in vitro regeneration, paving the way for further research into the potential of adaptogenic plants to modulate plant stress in a manner similar to their effects in humans and animals. Moreover, the role of TRM and NAS in enhancing regeneration demonstrates their significance as regulatory molecules. Collectively, these observations highlight the importance of the synergistic interactions among compounds and underscores the necessity of developing pathway-specific strategies to investigate complex plant responses.

## 4. Materials and Methods

### 4.1. Plant Materials and Tulsi Leaf Extract Preparation

Plants of the tulsi (*Ocimum sanctum*) line ‘Vrinda’ [79] maintained in the medicinal plant collection at the Gosling Research Institute for Plant Preservation (GRIPP) were used for this study. The plants were grown and agronomically maintained in a greenhouse environment (23 °C and 45% relative humidity) at the University of Guelph, ON, Canada. Briefly, the plants were grown in pots (6–8” diameter) filled with Sunshine professional growing media (Sun Gro Horticulture, Vancouver, BC, Canada), and watering occurred once every 3 days. Fully grown mature plants at the flowering stage were used to collect the leaves for extraction.

Healthy leaves were collected and dried at a temperature of 55 °C in a drying room for 3 days. Air-dried samples weighing 10 g were grounded into a fine powder using a mortar and pestle, and an aqueous extract was prepared by heating the samples (75 °C) in 1 L of double-distilled water for 30 min. The extract was then filter-sterilized (0.2 μm pore size) using a Welch vacuum (Ideal vacuum, Albuquerque, NM, USA) in a laminar flow hood and stored in the dark at room temperature until further use.

### 4.2. N. tabacum Culture Establishment and Leaf Explant Culture

*N. tabacum* plant material was obtained from the GRIPP medicinal plant collection (University of Guelph, Guelph, ON, Canada) and was multiplied using nodal explants cultured in Magenta boxes (Magenta Corporation, Chicago, IL, USA) containing Murashige and Skoog (MS) basal medium with MS vitamins [80] supplemented with 3% sucrose. The pH adjusted to 5.7 before solidifying with 0.22% Phytagel (Sigma Aldrich, Oakville, ON, Canada). The medium was sterilized by autoclaving at 121 °C for 20 min. The cultured plants were maintained at 25 °C under “cool white” fluorescent tubes (Osram Sylvania Ltd., Mississauga, ON, Canada) at a 20–25 μmol m^−2^ s^−1^ intensity in a 16 hr photoperiod. Fully expanded young leaves from 3-week-old nodal cultures were used as the explants.

The leaves were cut into 0.8 cm circles using a cork borer, avoiding the main veins, and these explants were cultured in Petri dishes (100 × 25 mm) containing 20 mL of MS medium supplemented with 3% sucrose, 2.0 μM 6-benzylaminopurine (BA), and 0.2 μM 1-naphthaleneacetic acid (NAA) added prior to autoclaving and 20% tulsi leaf extract added after autoclaving. The cultures were incubated at 25 °C under cool white light conditions (20–25 μmol m^−2^ s^−1^;16 h photoperiod). Control plates were prepared and maintained similarly without adding tulsi leaf extract.

Thirty explants (5 per plate) were used to test the treatment effect, and all the experiments were repeated twice. All the primary cultures were examined daily for the effects of the extracts on the growth and differentiation of shoots and somatic embryos. After 4 weeks in culture, shoot organogenesis and somatic embryogenesis induction were investigated and compared between the control and 20% tulsi leaf extract-treated explants. Regeneration was quantified by recording organogenic shoots greater than 1 cm in size and somatic embryo-like structures at the globular or more developed stages. Uniform-sized explants from additional plates of all the treatments were collected on days 0, 10, and 25 in culture. The medium was cleaned from the explants with a paper towel, and the samples were kept in the freezer at −80 °C until biochemical analysis.

### 4.3. Histology of Shoot Organogenesis and Somatic Embryogenesis

To study the ontogeny and development of *N. tabacum* shoots and embryo-like structures, the leaf explants were collected after 5, 10, 15, and 25 days in culture, and leaf sections were fixed in formaldehyde and acetic acid in an alcohol medium (1:1:18 (*v*/*v*/*v*) FAA: 37% (*w*/*v*) formaldehyde; 50% (*v*/*v)* acetic acid, and 95% (*v*/*v)* ethanol) at room temperature for 48 h. The samples were then transferred to 70% (*v*/*v*) ethanol for storage until analysis and were then dehydrated through an incremental ethanol series (50, 70, 85, 95%) and embedded in paraffin [81]. Longitudinal sections were made (10 μm thick) using a paraffin-compatible microtome (Leica RM2255, Leica Biosystems, Deer Park, IL, USA). The sections were stained using toluidine blue (0.01%) and observed under a light microscope (B120 binocular compounds microscope, AmScope, Irvine, CA, USA).

### 4.4. Detection and Quantification of Indoleamine Metabolites

#### 4.4.1. Sample Collection and Extraction

Indoleamine metabolites were detected and quantified using ultra-performance liquid chromatography–mass spectrometry (UPLC-MS). A slightly modified methanol double extraction protocol described by Ayyanath et al. [82] was followed to prepare the samples. To obtain leaves for the analysis of indoleamines on day 0, intact and cut leaves were utilized. For the intact leaves, the entire plants from 3-week-old nodal cultures were submerged into liquid nitrogen, and the fully expanded young leaves were separated. Leaves from plants of additional cultures of the same age were collected and cut into discs using a cork borer and immediately frozen in liquid nitrogen.

In addition to day 0, leaf samples containing developing regenerative structures from days 10 and 25 were also harvested and finely powdered. The powdered samples (100 mg) were extracted using 500 µL of cold extraction solvent comprised of 75% UPLC-MS-grade methanol and 5% formic acid (Fisher Chemical, Ottawa, ON, Canada) in Milli-Q water. The mixture was vortexed and kept for 1 h at −20 °C and was then spun down at 14,000 rpm at 4 °C for 15 min (Sorvall ST 8R, ThermoFisher Scientific, Dreieich, Germany). The supernatant was collected and kept at −20 °C until the second extraction was completed. A second extraction was performed on the same sample using similar conditions described above, and the supernatants were pooled. The supernatant was then purified and preconcentrated using the solid phase extraction (SPE) technique described by Yalçın et al. [83].The metabolites retained in the SPE cartridge (Oasis^®^ HLB, 1 cc, Waters, MA, USA) were eluted with 200 μL methanol and were then filtered through a 0.22 µM centrifuge filter (Millipore, Darmstadt, Germany; 1 min, 13,000 rpm). The flow-through was immediately analyzed using UPLC-MS.

#### 4.4.2. Separation and Quantification of Metabolites

The metabolites were separated using a reverse-phase ultra-performance liquid chromatography system (UPLC: Shimadzu LabSolutions, MD, USA) by injecting 5 μL aliquots of sample into Shim-pack Scepter LC column (2.1 × 50 mm, 1.9 µm; Mandel Scientific Company, Guelph, ON, Canada). The metabolites were separated using a gradient of solvents A (0.1% formic acid, pH 3.0) and B (100% acetonitrile) with initial conditions of 95% A (5% B) increased to 5% A (95% B) over 4 min using a curve of 0. The column temperature was 40 °C, and the flow rate was 0.2 mL min^−1^.

The metabolites TRP, TRM, 5-HTP, SER, NAS, MEL, 2-OHMEL, and AFMK were detected in the positive mode for a mass-to-charge ratio (*m/z*) of 205, 161, 221, 177, 219, 233, 249, and 265, respectively using a single-quadrupole mass spectrometer (LCMS 2020, Shimadzu, Kyoto, Japan) in the single ion recording mode (SIR). All the standards were analytical grade and purchased from Sigma Aldrich, Oakville, ON, Canada. The instrument probe temperature was set to 250 °C with a gain of 5, and the capillary positive and negative voltages were set to 0.5 kV. These metabolites were compared to their respective standards (UPLC-MS grade) and quantified using a standard curve generated using a similar separation method and gradient conditions. Data from two biological and two technical replicates were used for the calculations (LabSolutions, v5.109/2020, Shimadzu Corporation, Kyoto, Japan).

### 4.5. Statistical Analysis

The experiment was designed in a completely randomized way, with the treatment being the main factor of interest. The data were subjected to an analysis of variance (ANOVA) using PROC GLIMMIX in SAS^®^ Studio (SAS Institute Inc., Cary, NC, USA). The data were tested for normalization using Shapiro–Wilk normality tests, and the error assumptions of the variants were analyzed using homogenous and studentized residual tests. The significant differences between the control and 20% tulsi leaf extract at each time point were determined using Tukey’s honest significant different (HSD) multiple comparison tests (α = 0.05). The means ± SE for each metabolite and regeneration type were presented in graphical and tabular formats, respectively.

## Figures and Tables

**Table 1:** The effects of tulsi leaf extract on the induction of shoots and somatic embryo-like structures from cultured leaf discs of *N. tabacum*. The numbers of regenerants per explant including shoots and embryo-like structures represent the average of 30 explants.

Treatment	Average Number of Regenerants per Explant	Average Number of Shoots Greater than 1 cm per Explant	Average Number of Embryo-like Structures
Control	6.14 ± 0.5 ^b^	6.14 ± 0.5 ^b^	0 ^b^
Tulsi leaf extract (20%)	13.07 ± 0.5 ^a^	12.05 ± 0.5 ^a^	1.53 ± 0.1 ^a^

Values represent means ± SE, and different letters within a column represent significant differences between the treatments based on Tukey’s HSD test at α = 0.05.

## Data Availability

The authors confirm that the data supporting the findings of this study are available within the article.

## Supplementary Materials

The following supporting information can be downloaded at: https://www.mdpi.com/article/10.3390/plants13101370/s1, Table S1: Preliminary study using various tulsi leaf extract concentrations on in vitro regeneration; Table S2: Adj. *p* values of indoleamines at various developmental stages; Figure S1. Indoleamine pathway schematic.
