# Supplementary material for: Enhanced In Vitro Plant Morphogenesis of Tobacco: Unveiling Indoleamine-Modulated Adaptogenic Properties of Tulsi (Ocimum sanctum L.)
Source: Plants (Basel). 2024 May 15;13(10):1370. doi: 10.3390/plants13101370 (PMC11125241; doi:10.3390/plants13101370)
Supplement: Supplementary file 1 [file plants-13-01370-s001.zip › plants-3003201-supplementary.pdf]

Table S1. The effect of tulsi leaf extracts on the induction of regenerants, including organogenic shoots and somatic embryo-like structures in *N. tabacum* leaf explants. Values represent means ± SEM, and different letters within the same column represent significant differences between treatments using the Tukey-HSD test (α = 0.05). P-values relative to the control treatment to determine if differences between the extract and control treatments were statistically significant.

**Treatment**	**Average number of regenerants greater than 1 cm per explant**	**Adj.p (relative to control)**
Control	6.14 ± 0.5 c	
1% Tulsi leaf extract	9.60 ± 0.5 bc	<0.0001
10% Tulsi leaf extract	12.58 ± 0.5 ba	<0.0001
20% Tulsi leaf extract	13.07 ± 0.5 a	<0.0001

Table S2. Adj P-values of various control and tulsi leaf extract treatments compared at day 10 and 25 *in vitro*.

**Treatment_day**	**Adj.p values (Fresh weight concentration)**						
	TRP	TRM	5-HTP	SER	NAS	MEL	2-OHMEL
C_10 vs 20% hb_10	0.9477	<.0001	0.7208	0.0527	<.0001	0.9942	0.004
C_25 vs 20% hb_25	1.0000	0.1535	0.1414	0.9717	0.2209	0.9986	0.6689

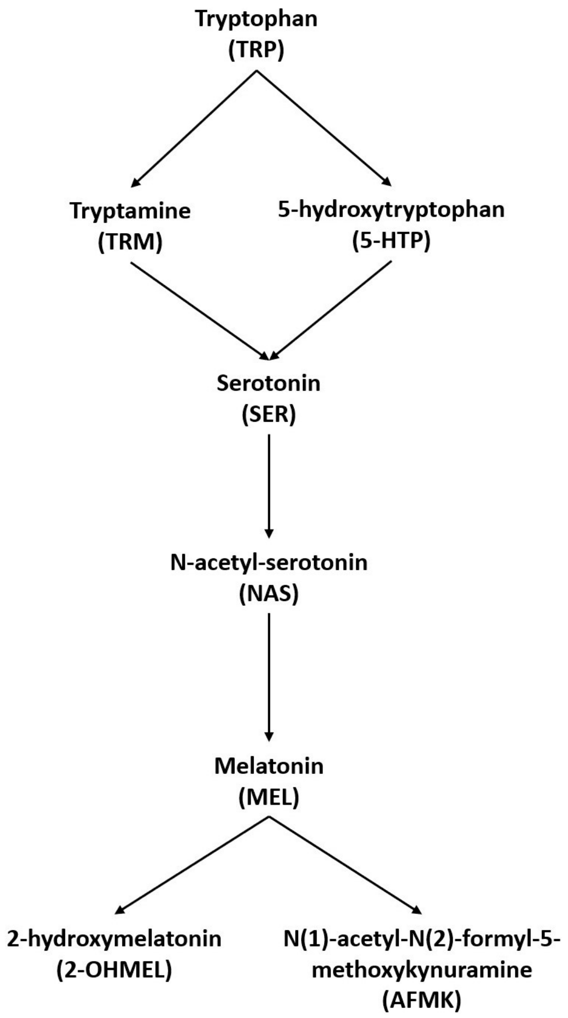

Figure S1. Schematic representation of the indoleamine pathway beginning with the precursor molecule tryptophan and ultimately converting into the downstream molecules 2-hydroxymelatonin and N(1)-acetyl-N(2)-formyl-5-methoxykynuramine [30,57].
